# Recidivism rates in individuals receiving community sentences: A systematic review

**DOI:** 10.1371/journal.pone.0222495

**Published:** 2019-09-20

**Authors:** Denis Yukhnenko, Achim Wolf, Nigel Blackwood, Seena Fazel

**Affiliations:** 1 Department of Psychiatry, University of Oxford, Oxford, United Kingdom; 2 Institute of Psychiatry, Psychology and Neuroscience, King’s College London, London, United Kingdom; Geneva University Hospitals, SWITZERLAND

## Abstract

**Objective:**

We aimed to systematically review recidivism rates in individuals given community sentences internationally. We sought to explore sources of variation between these rates and how reporting practices may limit their comparability across jurisdictions. Finally, we aimed to adapt previously published guidelines on recidivism reporting to include community sentenced populations.

**Methods:**

We searched MEDLINE, PsycINFO, SAGE and Google Scholar for reports and studies of recidivism rates using non-specific and targeted searches for the 20 countries with the largest prison populations worldwide. We identified 28 studies with data from 19 countries. Of the 20 countries with the largest prison populations, only 2 reported recidivism rates for individuals given community sentences.

**Results:**

The most commonly reported recidivism information between countries was for 2-year reconviction, which ranged widely from 14% to 43% in men, and 9% to 35% in women. Explanations for recidivism rate variations between countries include when the follow-up period started and whether technical violations were taken into account.

**Conclusion:**

Recidivism rates in individuals receiving community sentences are typically lower in comparison to those reported in released prisoners, although these two populations differ in terms of their baseline characteristics. Direct comparisons of the recidivism rates in community sentenced cohorts across jurisdictions are currently not possible, but simple changes to existing reporting practices can facilitate these. We propose recommendations to improve reporting practices.

## Introduction

Prison populations and their associated costs continue to grow worldwide [1, 2, 3]. Community sentences, which are in part designed to limit the prison population, are therefore of considerable interest to policy makers in the criminal justice system. The community sentenced population is also significantly larger than the prison population. For example, in the US in 2013, there were 1.8 times more individuals placed on probation than incarcerated [4], and in the UK, the number of individuals who received a court order or a suspended sentence was 1.7 times higher than the number sentenced to prison [5]. Community sentences are a heterogeneous group of criminal justice disposals, which include suspended custodial sentences, probation with supervision, electronic monitoring, mandatory community service, mandatory treatment or training programmes, and other measures. Many experts consider that such community sentences are a cost-effective alternative to prison [6]. It is also suggested that they are more effective in reducing recidivism [7] and in preventing further criminalization of offenders [8, 9, 10].

To assess the impact of community sentences on recidivism, different methods to account for confounding, including the choice of matched controls, can be employed. This is important for validity, since direct comparisons of cohorts receiving different sentences are often complicated by selection biases, such as the likelihood of higher risk individuals to receive longer sentences. To account for potential selection biases, some studies have utilised the randomised allocation of sentenced individuals to an alternative sanction [11], different judges [12, 13], and different training programmes [14]. However, conducting randomised trials is often not possible in a criminal justice setting. Another approach to address confounding is to compare matched cohorts of individuals receiving different sentences. Propensity score matching, which is widely used, is based on predicted probability to reoffend [15, 16, 17, 18], whereas precision/exact matching is based on a set of predetermined individual characteristics [15]. They may also be used in combination [19]. Finally, there is a relatively new approach of using machine learning algorithms to sample stratification and allocation of individuals [20].

Although randomised controlled trials and matched cohort designs will generate more robust findings, they are often costly, and it is often not possible to utilise these methods when comparing the impact of systemic approaches to community sentencing on recidivism across different jurisdictions (especially between countries) because of different methods of data acquisition and legal thresholds for community sentences. The standardization of reporting practices will allow the comparisons based on routinely reported data to be as close to matched cohort designs as possible. In the previous review conducted by our research group, we demonstrated that, for national samples of released prisoners, such comparisons are currently difficult due to differences in reporting practices, definitions of outcomes, and follow-up period length [21]. Reporting recidivism in community sentenced populations may present additional challenges because, besides reconviction, rearrest and reimprisonment, recidivism reports for individuals receiving non-custodial measures may utilise other types of outcomes and follow-up schemes.

In order to provide a current picture of the recidivism information in community sentenced populations and to examine reporting practices, we sought to systematically review studies of recidivism rates in individuals receiving community sentences, to examine possible explanations for variation in such rates and to revisit our reporting guidelines to take into consideration the community sentenced population.

## Methods

The review protocol was registered in PROSPERO (CRD42018088156). We followed the design of the previous review of recidivism rates among released prisoners conducted by our research group [21]. We searched MEDLINE, PsycINFO and SAGE publication databases using search terms in relation to community offenders and recidivism (see S1 Table for search terms). In addition, Google Scholar and Google Web were used for targeted searches for the 20 countries with the largest prison populations worldwide [22]. We also reviewed the reference lists of the included publications.

We included cohort studies of the general population of adult offenders receiving community or suspended sentences with no follow-up period restrictions. If there were multiple reports for one country, we used national data from the most recent report. Regional data (i.e. reports from provinces, states or cities) were used when national data were unavailable. Authors of the studies were contacted when necessary.

We excluded studies that focused on specific subpopulations (e.g., sex offenders, mentally disordered offenders). We also excluded studies of individuals on parole after serving a prison sentence and of individuals specifically sentenced to undergo mental health and/or substance abuse treatment. Heterogeneous samples that also contained released prisoners or adolescents were excluded. In addition, we excluded matched cohort studies that compared community sentenced individuals with individuals released from prison, as matched cohorts were not representative of the general population.

We extracted information on the rates of general recidivism, violent recidivism, non-violent recidivism, and violation of probation conditions after the imposition of a community sentence. No outcome measurement restrictions were applied. Data extracted included country or territory, year of selection, index offence disposal, follow-up period, reported outcomes, base rates of reported outcomes. DY carried out the initial search on the date specified, using databases and search strategy listed above, and screened titles and abstracts. In addition, DY and AW carried out a targeted search of recidivism reports using governmental websites of the countries of interest. Quality assessment of included studies was conducted by DY using the NIH Quality Assessment Tool for Observational Cohort and Cross-Sectional Studies [23]. Any uncertainties were discussed with SF. Potential differences between the recidivism rates of men and women were examined using relative risk ratios, calculated according to Altman, 1991 [24]. No meta-analysis was conducted because of high heterogeneity in sample compositions and outcome definitions across included studies.

PRISMA guidelines [25] were followed (S1 and S2 Tables).

## Results

**Fig 1:**
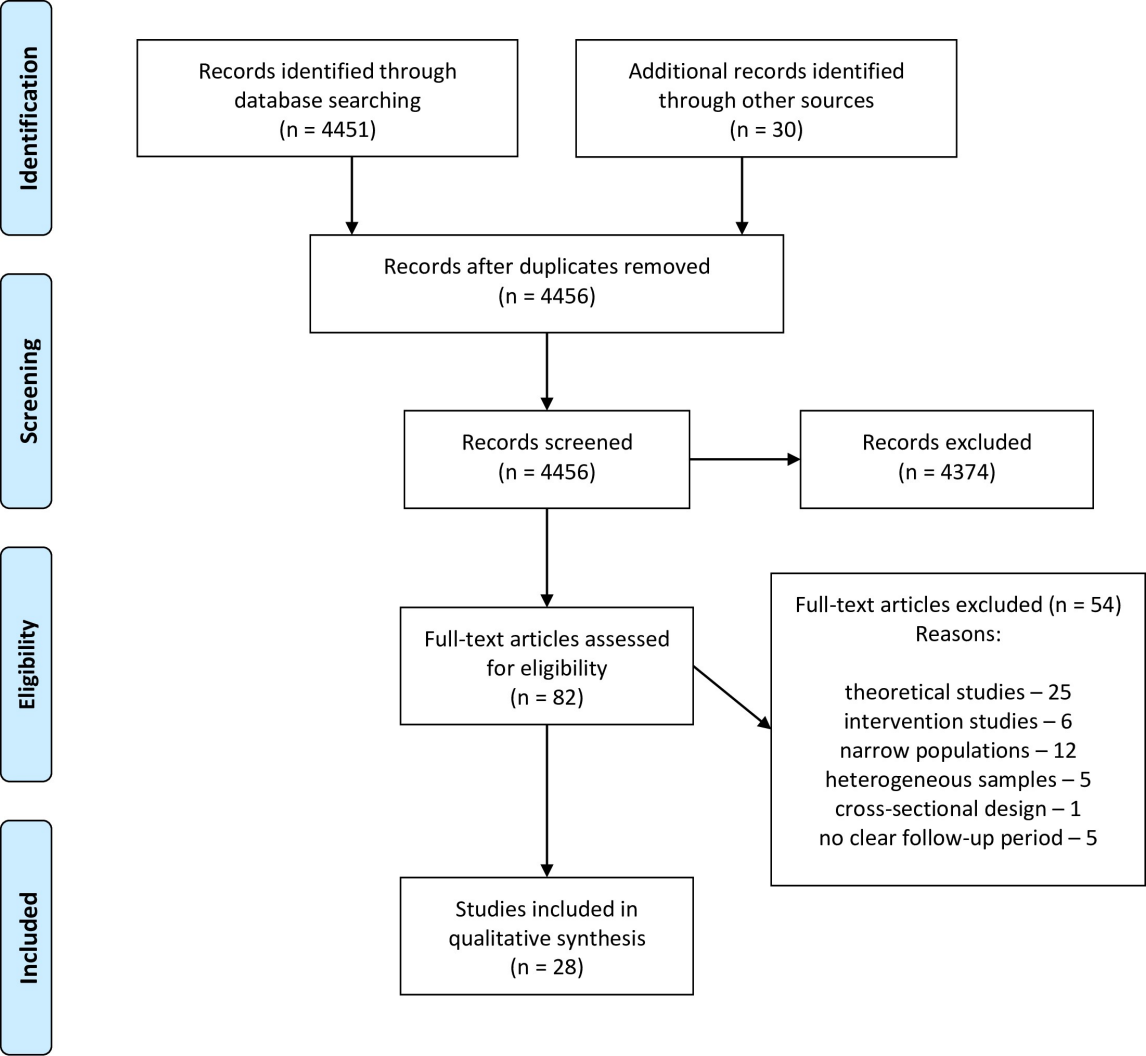
PRISMA 2009 flow diagram.

**Fig 2:**
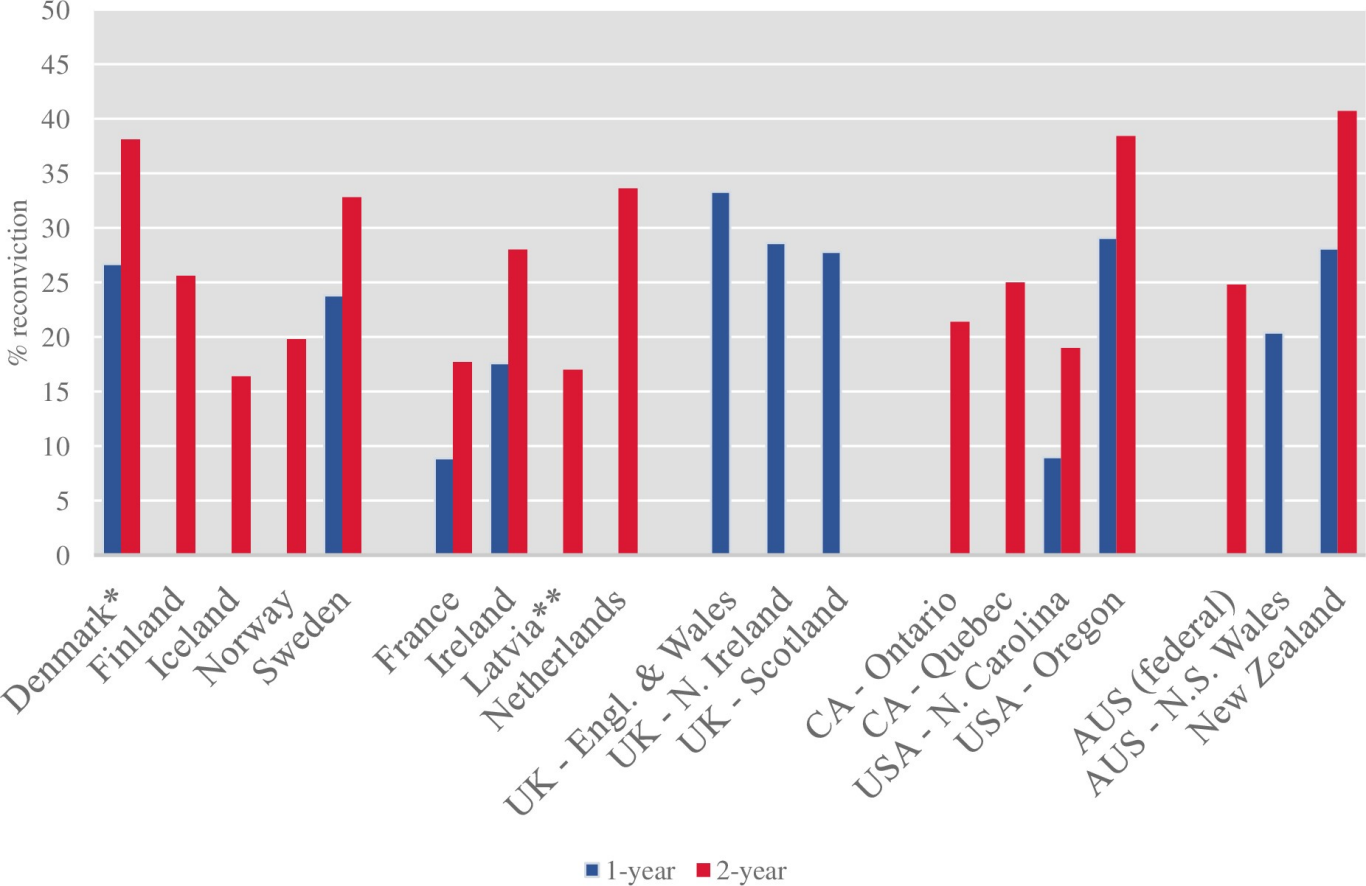
Reconviction rates in adult individuals receiving community sentences for 1-year and 2-year follow-up periods. For sources, refer to S3 Table. *The reconviction rate for Denmark reported for individuals aged 20 and older. All other cohort include individuals aged 18 and older. **Follow-up for Latvia was 29 months.

We identified 28 studies reporting recidivism rates in individuals receiving a community sentence from 19 countries (Fig 1, S3 Table). Of the 20 countries with the largest prison populations, recidivism reports were identified for the USA and UK. The data were mostly reported by governmental agencies; however, five identified papers were published in journals [12, 26, 27, 28, 29]. The results are provided separately for reconviction (Fig 2; Tables 1 and 3) and rearrest rates (Tables 2 and 3) for follow-up periods of one, two, three, five, and seven years. Only 11 identified reports and studies reported recidivism rates separately for men and women (Table 3).

**Table 1:** Reported reconviction rates for cohorts of adult individuals receiving community sentences.

			Length of follow-up period (years)				
Country	Selection period	Cohort size	1	2	3	5	7
**Europe**							
**Nordic countries**							
Denmark* [30]	2013	6,501	26.7	38.1			
Finland [31]	2005	3,767		25.6			
Iceland [31]	2005	73		16.4			
Norway [31]	2005	2,839		19.8			
Sweden [32]	2008	22,306	23.8	32.8	38.1		
**The United Kingdom**							
England and Wales [33]	2015/2016	139,617	33.3				
Northern Ireland [34]	2014/2015	6,234	28.6				
Northern Ireland [35]	2005	4,425		26.1			
Scotland [36]	2014/2015	21,733	27.8				
**Other**							
France [37]	2004	241,996	9.1	18.1	25.2	34.3	
Germany [38]	2007	96,521			39.0		
Italy [29]	1998	8,817					19.0
Ireland, Republic of [39]	2010	3,698	17.6	28.0	36.4		
Latvia** [28]	2009	1,190		17.0			
Netherlands [40]	2003	38,530		33.6			
**North America**							
**Canada**							
Ontario [41]	2013/2014	35,561		21.4			
Quebec [42]	2007/2008	4,851		25.0			
**USA**							
Michigan [12]	2003/2006	43,606	5.5		17.9	24.9	
North Carolina [43]	2013	35,103	9.0	19.0			
New York State [44]	2002	31,267				33.0	
Oregon [45]	2014	4,403	29.1	38.4	43.8		
**South America**							
Chile [46]	2007	23,736			27.7		
**Oceania**							
**Australia**							
Australia (federal) [47]	2012/2013	n/a		24.8			
New South Wales [48]	2015	16,907	20.4				
**New Zealand**							
New Zealand (federal) [49, 50]	2013/2015	n/a	28.1	40.7			

*The reconviction rate for Denmark reported for individuals aged 20 and older. All other cohort include individuals aged 18 and older.

**Follow-up for Latvia was 29 months.

**Table 2:** Reported rearrest rates for cohorts aged 18 and older.

			Length of follow-up period (years)					
Country	Selection period	Cohort size	1	2	3	4	5	7
**North America**								
**USA**								
USA (federal) [27]	2004/2005	13,504	13.1	22.2	29.0	34.6	38.8	44.9
Illinois [51]	2006	2,770					54.2	
North Carolina [43]	2013	35,103	26.0	38.0				
Oregon [45]	2014	4,403	34.8	44.7	50.4			
**South America**								
Chile [46]	2007	23,736			40.6			

**Table 3:** Reconviction and rearrest rates in adult men and women receiving community sentences.

Country	Selection period	Follow-up		Sample size	%	Relative risk (95% CI)
***Reconviction rates***						
**Europe**						
**Nordic countries**						
Denmark* [30]	2013	1 year	Men Women	5,413 1,088	32.6 18.2	1.80 (1.57–2.04)
Denmark* [30]	2013	2 years	Men Women	5,413 1,088	40.7 25.2	1.61 (1.45–1.80)
**The United Kingdom**						
Northern Ireland** [35]	2005	1 year	Men Women	16,233 2,814	20.8 10.4	2.00 (1.78–2.24)
**Other**						
Ireland, Republic of [39]	2010	1 year	Men Women	3,241 457	18.0 14.7	1.23 (0.97–1.55)
**North America**						
**Canada**						
Quebec [42]	2007/2008	2 years	Men Women	4,010 830	26.0 21.0	1.24 (1.08–1.43)
**USA**						
North Carolina [43]	2013	2 years	Men Women	25,850 9,253	21.0 14.0	1.50 (1.42–1.59)
Oregon [45]	2015	1 year	Men Women	3,102 1,231	27.6 27.0	1.02 (0.92–1.14)
Oregon [45]	2014	2 years	Men Women	3,200 1,203	39.8 34.6	1.15 (1.05–1.26)
Oregon [45]	2014	3 years	Men Women	3,200 1,203	45.5 39.3	1.16 (1.07–1.25)
**South America**						
Chile [46]	2007	3 years	Men Women	20,399 3,337	27.4 27.8	0.99 (0.93–1.05)
**Oceania**						
**Australia**						
Australia (federal) [47]	2012/2013	2 years	Men Women	n/a	43.4 31.1	n/a
New South Wales [48]	2015	1 year	Men Women	13,744 3,163	20.9 18.1	1.15 (1.06–1.25)
Western Australia [47]	2012/2013	2 years	Men Women	n/a	13.9 9.3	n/a
**New Zealand**						
New Zealand (federal) [49]	2014/2015	1 year	Men Women	n/a	30.0 21.4	n/a
***Rearrest rates***						
**North America**						
**USA**						
Illinois [51]	2006	5 years	Men Women	2215 542	55.9 47.2	1.18 (1.07–1.30)
North Carolina [43]	2013	2 years	Men Women	25,850 9,253	41.0 29.0	1.41 (1.37–1.46)
Oregon [45]	2015	1 year	Men Women	3,102 1,231	34.2 34.2	1.00 (0.91–1.10)
Oregon [45]	2014	2 years	Men Women	3,200 1,203	46.7 39.3	1.19 (1.10–1.29)
Oregon [45]	2014	3 years	Men Women	3,200 1,203	52.7 44.2	1.19 (1.11–1.28)

Note: relative risk ratios were calculated according to Altman (1991)[24]. Reported data for New Zealand, Australia (federal, New South West and Western Australia) did not allow for relative risk calculation.

* The reconviction rate for Denmark reported for individuals aged 20 and older.

** Data by gender for Northern Ireland are reported for all non-custodial disposals (including fines).

**Fig 3:**
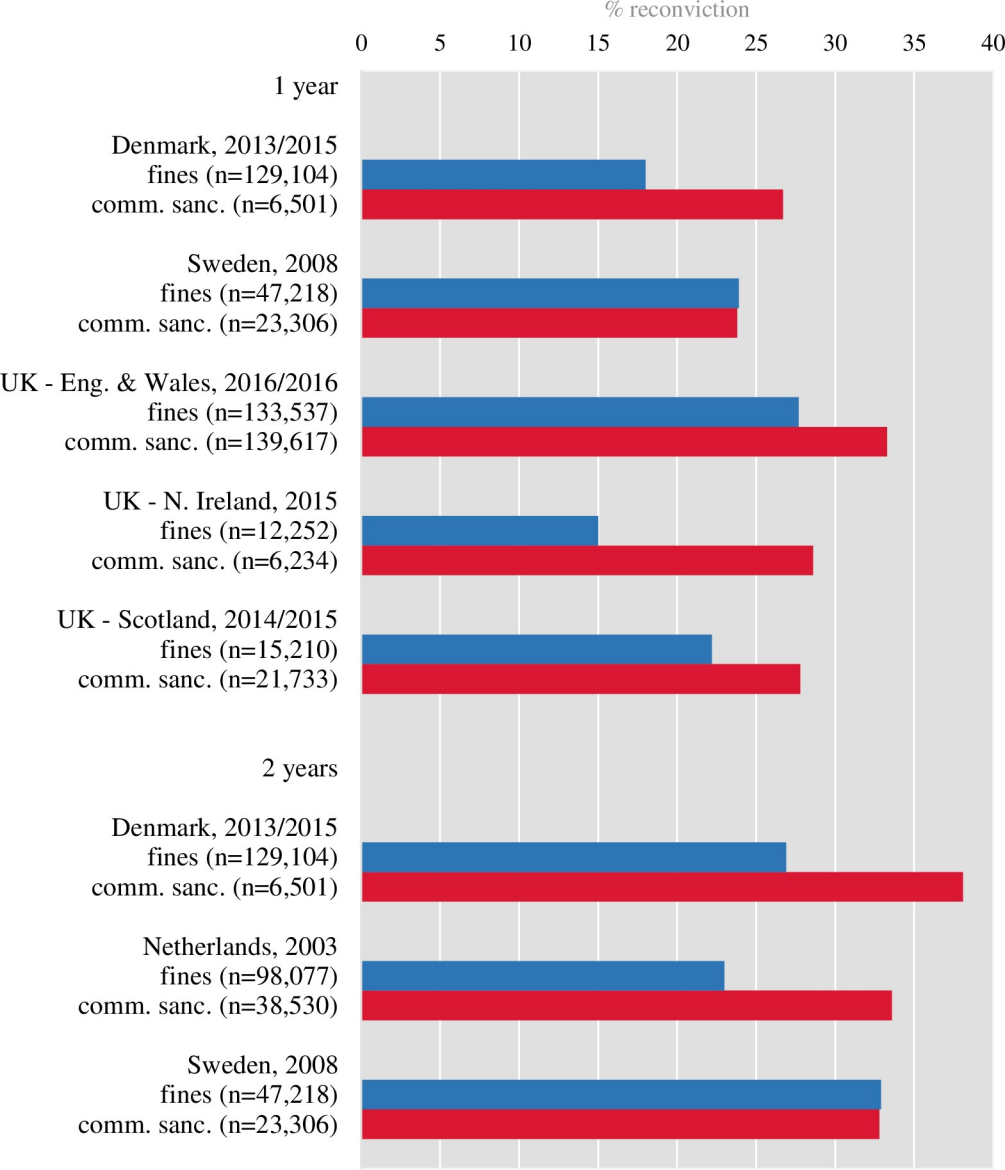
Comparison of reconviction rates in adult individuals receiving fines or sentenced to other community sanctions for 1-year and 2-year follow-up periods. For Denmark, the data reported is only for fines larger than DKK 2,500 for road traffic offences and DKK 1,000 for most other offences (only individuals aged 20 and older at the time of offence are included in the cohort). In Scotland, when an individual receives more than one type of sentence (i.e., community supervision and fine), only the most serious sentence is accounted for, which is not the case in other countries.

The composition of the identified cohorts varied in terms of the sentencing and demographic characteristics of included offenders. Many published reports were excluded because they used heterogeneous samples of offenders such as released prisoners and probationers, or adolescents and adults, without providing recidivism rates for the subgroups (e.g., [52]). Equally, we excluded reports that provided recidivism statistics using cross-sectional data (e.g., [53]). Several reports included fines as a community sanction and their inclusion influenced reported recidivism rates (Fig 3).

Most of the included studies used representative cohorts of adult offenders (aged 18 and older) receiving a community sentence. However, the Danish data were for offenders aged 20 and older (since the lower age tier was 15–19 years old), and the Republic of Ireland data did not include sex offenders (see S4 Table). Several included reports and studies (from Latvia, New Zealand and Australia) did not provide cohort sizes, and they could not be estimated from other sources.

The recidivism outcome and its operationalisation varied significantly between countries and even between territories within one country (see S4 Table). The most commonly used outcome was reconviction with a follow-up period of one or two years. To register a reconviction, some countries allowed additional time after a declared follow-up period for the offence to be proven in court (e.g., England and Wales, Northern Ireland, Sweden, and others). Other countries used the initiation of legal proceedings, if there was no acquittal, as an outcome event (e.g., Latvia, the Netherlands). In addition, a follow-up period could start at the beginning or at the end of a community sentence. In the latter case, a violation of probation conditions was not counted as a recidivism event. It was often unclear how offenders that had received multiple sanctions for one offence were counted in the reports. Some reports considered each sanction separately (and thus one offender could be counted several times), while others only included the most serious sanction for one offender. Other reports did not clarify this matter. Several reports on probation outcomes (from Australia, USA, and Singapore) were excluded from the analysis, as they did not have fixed follow-up periods and only followed offenders during the length of the sentence (which may have varied).

The reported general recidivism rates varied significantly between countries and regions (Fig 2; Tables 1 and 2). For example, the one-year reconviction rate ranged between 5.5% in Michigan, USA to 33.3% in England and Wales. Two-year reconviction rates varied from 16.4% in Iceland to 40.7% in New Zealand. Rearrest rates were reported by several US states with notable differences (22% for federal probationers to 45% in Oregon during 2-year follow-up).

In addition to general recidivism, several countries reported specific recidivism (committing a new crime similar to an index offence) and only two (Denmark and Republic of Ireland) provided data that allowed for calculation of different types of recidivism for community sanctioned populations based on the type of new offence. For example, in the Republic of Ireland, reconviction for violent crimes (we included homicides, sexual offences, attempts/threats of murder, assaults, harassments, kidnapping, robbery/extortions) accounted for only 5% of all recidivism cases. In Denmark, violent crimes also included crimes against public order and constituted 11% of all reconviction cases.

Of all the included reports and studies, only reports from Quebec [42], North Carolina [43] and Michigan [12] separately reported data on technical violations of community sentences. In North Carolina, technical violation automatically led to up to 90 days of imprisonment, whereas in Quebec a breach did not necessarily lead to incarceration. In Michigan, reimprisonment after technical violation was examined as a separate outcome.

Only 10 identified reports and studies provided recidivism data separately for men and women (Table 3). Most of them indicated that female offenders had a lower risk of recidivism than male offenders, but with noticeable variability. For example, the absolute risk difference for one-year reconviction rates varied between 0.6% in Oregon, USA and 17.4% in Denmark.

## Discussion

In the present study, 28 reports were identified from 19 countries that reported reoffending rates in individuals given community sentences at the point of court disposal. Only two of these were from the 20 countries with the largest prison populations. Overall, reported 1-year reoffending rates varied between 5% and 33%, and 2-year rates ranged from 16% to 41%. Such recidivism rates are lower in comparison to those observed in released prisoners [21]. In addition, women in general have a lower risk of recidivism when serving a community sentence than men. This is consistent with findings in released prisoners [54]. Many studies were excluded from the review because they reported recidivism rates only for released prisoners or for a mixed sample of individuals who received community and custodial sentences. Although community sentenced individuals constitute the largest population of those receiving criminal sanctions, this review suggests that reporting practices concerning their outcomes need improvement in terms of coverage and detail.

To examine our findings further, we have undertaken a structured search in the last 5 years of studies in offenders under community supervision with randomised or matched control groups (S5 Table). We found that community sentences were typically associated with lower recidivism rates than custodial sentences, particularly in those individuals with low to moderate risk levels. In addition, this overview shows that specialised community programmes is more likely to benefit individuals in certain offender groups (such as DWI offenders) in relation to reoffending outcomes. Another finding from these research studies is that they were influenced by the type of model used for matching and the definition of outcome (re-arrest vs reconviction vs reimprisonment).

Overall, the wide variation in recidivism rates between countries was expected. The same factors play a role with individuals given community sentences as with released prisoners, such as differences in judicial practices, and definitions and operationalisations of recidivism measures. Variations in laws and precedent practices across jurisdictions results in cohorts with different proportions of individuals convicted for different types of offences. For example, a conviction for the same drug-trafficking offence may lead to a suspended sentence or probation in one jurisdiction or to 5–10 years in prison in another [55]. Because of this variability, sentenced cohorts may not be equivalent in terms of their initial recidivism risk.

There are, however, some additional issues which contribute to recidivism rate variability in community offenders. First, the set of actual sanctions that comprise community sentences are different depending on the legal system. There are many different combinations of community service work, probation with supervision, treatment orders, suspended sentences, and fines. Community sentences may also include house arrest or night-time curfews. Different combinations of sanctions will lead to variations in recidivism rates. One notable example is the inclusion of fines (Fig 3). In Northern Ireland [35], the inclusion of fines as an index sanction decreased the reported 2-year reconviction rate from 26% to 19%. Moreover, countries use different rules for the inclusion of fines. In Sweden and the Netherlands, fines and compensatory orders issued by courts and the prosecution office, but not the police, are reported. In Northern Ireland, England and Wales, fines that results from court convictions are reported. In Scotland, the data for “monetary disposals” are reported, which only includes fines issued by courts. Often, the operational definitions of sanctions are not given and recidivism data are not reported by separate types of sanctions. Providing recidivism data by sanction type may allow comparison between groups of similarly sentenced individuals and in turn substantially increase overall comparability between reports.

Second, there are many different approaches to defining the starting point of a follow-up period in individuals given community sentences. In many reports, the distinction between a follow-up period and a period of supervision was not always clearly described and accounted for. For example, a follow-up period might start after the completion of community service, i.e. after the end of supervision. This approach ignores a significant amount of time when a sentenced individual is at risk of committing a new offence. This may also lead to underreporting of recidivism because offenders who have violated the conditions of community sentences during the time of supervision, and subsequently been incarcerated, are excluded from observation. Another definition for time at risk could be a follow-up period that matches a period of supervision. Some countries and regions (e.g., New York State) may have a fixed period of supervision (5 years) that matches the period of follow-up, and reported recidivism rates in this case are virtually the same as sentence completion rates. If there is no set period for supervision and only completion rates are reported, then the follow-up time period is unclear. In addition, the follow-up period may start with the beginning of supervision, partially overlap with it and extend beyond it. The clear separation of sentence completion rates and post-supervision recidivism rates will enable improved between-jurisdiction comparisons. The report from Quebec [42] illustrates the importance of distinguishing between sentence completion and reconviction during a follow-up, since reported recidivism rates vary from 25% to 41% depending on the starting point employed. There is also a qualitative distinction between recidivism under supervision and recidivism during unsupervised community living, which is important to take into account, especially given the fact that supervision periods may vary between offenders. In the context of community sentences, additional reporting of the rate of technical violations and reconvictions based on technical violations is important for international comparisons, since the handling of such violations by courts and probation systems has significant impact on outcomes.

Finally, there may be differences in the quality of supervision, availability and effectiveness of rehabilitation programmes, availability of vocational training, and access to healthcare services. These are factors that researchers and practitioners are keen to examine; however, because of differences in reporting practices, it is difficult to compare these factors internationally.

Reporting practices can be improved to facilitate international comparisons and to help to inform sentencing decisions. The general recommendation for recidivism reporting in any population is to provide a detailed breakdown of the sample by index offence type, socio-demographic characteristics and outcomes, which may include general and violent recidivism. We additionally propose two main recommendations specific to reporting recidivism in community sentenced populations. First, recidivism data should be additionally reported by types of sanctions (i.e. community service, electronic monitoring or mandatory treatment) with clear operational definitions of sanction types provided. Second, the completion rate and recidivism rate after the completion of supervision should be reported separately with a clear indication of supervision and follow-up lengths (this could also be applied to released prisoners on parole). Following these recommendations will enable flexible extraction of recidivism data for more meaningful comparisons across jurisdictions.

## Strengths and limitations

To the authors’ knowledge, this is the first study to systematically review recidivism rates in a general adult population of individuals receiving community sentences. Studies included in the review were generally of high quality (as assessed by the NIH Quality Assessment Tool for Observational Cohort and Cross-Sectional Studies) and were conducted using large samples.

The heterogeneity of sample compositions and outcome definitions did not allow for direct quantitative comparisons of recidivism rates between different studies. However, we were able to examine how implementation of certain reporting practices influences the reported recidivism rates and what changes could be made to ensure that recidivism data is more comparable internationally in the future.

## Conclusions

We conclude that recidivism rates in the community sentenced population vary considerably between countries, and, in most cases, different criteria for time at risk and outcome are used. The recidivism rates are lower in comparison to those observed in released prisoners [21]. The comparability of recidivism rates can be improved if more detailed information is provided, and completion rates and recidivism after the end of supervision reported separately. The identified methodological problems specific to recidivism reporting in community sentenced populations can be addressed by adjustments to existing reporting practices. We have therefore published an updated version of the recidivism reporting checklist (S6 Table, [56]) which builds on our previous review of general criminal recidivism [21] to enable consistency and transparency in recidivism rate comparison between countries. We hope that this will enable the development of more informed policy and judicial decision-making in the criminal justice system.

## Supporting information

S1 Table Terms and search conditions used for systematic search in publication databases. (DOCX)

S2 Table PRISMA checklist. (DOC)

S3 Table Identified studies and reports that satisfied the inclusion criteria. Only reports containing the most recent data were included for a given territory or country. The data were mostly reported by governmental agencies; however, four identified papers were published in scientific journals (Bartels, 2009; Flores et al., 2017; Ķipēna et al., 2013; Leonardi, 2007). Several sources (Department of Correctional Services, 2014; Department of Corrections, 2016, 2017; Ķipēna, Zavackis, & Ņikišins, 2013) did not report cohort size. Data for Denmark and Oregon, USA were obtained by using online data tools within governmental agency websites. Quality assessment was conducted using NIH Quality Assessment Tool for Observational Cohort and Cross-Sectional Studies.
(DOCX)

S4 Table Description of data extracted from the studies. (DOCX)

S5 Table Recent studies in community sentenced populations that utilised advanced research designs. (DOCX)

S6 Table Recidivism reporting checklist. (DOCX)
