# Supplementary material for: Recidivism rates in individuals receiving community sentences: A systematic review
Source: PLoS One. 2019 Sep 20;14(9):e0222495. doi: 10.1371/journal.pone.0222495 (PMC6754149; doi:10.1371/journal.pone.0222495)
Supplement: S1 Table — (DOCX) [file pone.0222495.s001.docx]

**S1. Terms and search conditions used for systematic search in publication databases.**

| Search on MEDLINE, 1946 to March 16, 2018, and PsychINFO, 1806 to March 16, 2018, with no language restrictions: |
| --- |
| (recidivism OR reconviction OR reoffending) AND (USA OR “United States” OR China OR Russia* OR Brazil OR India OR Thailand OR Mexico OR Iran OR Indonesia OR “South Africa” OR Turkey OR Vietnam OR Colombia OR Philippines OR Ethiopia OR Ukraine OR “United Kingdom” OR England OR Poland OR Pakistan OR Morocco) AND (probation OR “community service” OR “community order” OR “community correction” OR “community sentence”) |
| Search on SAGE Journals, 1902 to March 16, 2018, with no language restrictions: |
| [[All recidivism] OR [All reconviction] OR [All reoffending]] AND [[All usa] OR [All "united states"] OR [All china] OR [All russia*] OR [All brazil] OR [All india] OR [All thailand] OR [All mexico] OR [All iran] OR [All indonesia] OR [All "south africa"] OR [All turkey] OR [All vietnam] OR [All colombia] OR [All philippines] OR [All ethiopia] OR [All ukraine] OR [All "united kingdom"] OR [All england] OR [All poland] OR [All pakistan] OR [All morocco]] AND [[All probation] OR [All "community service"] OR [All "community order"] OR [All "community correction"] OR [All "community sentence"]] |
