# Supplementary material for: Recidivism rates in individuals receiving community sentences: A systematic review
Source: PLoS One. 2019 Sep 20;14(9):e0222495. doi: 10.1371/journal.pone.0222495 (PMC6754149; doi:10.1371/journal.pone.0222495)
Supplement: S3 Table — Only reports containing the most recent data were included for a given territory or country. The data were mostly reported by governmental agencies; however, four identified papers were published in scientific journals (Bartels, 2009; Flores et al., 2017; Ķipēna et al., 2013; Leonardi, 2007). Several sources (Department of Correctional Services, 2014; Department of Corrections, 2016, 2017; Ķipēna, Zavackis, & Ņikišins, 2013) did not report cohort size. Data for Denmark and Oregon, USA were obtained by using online data tools within governmental agency websites. Quality assessment was conducted using NIH Quality Assessment Tool for Observational Cohort and Cross-Sectional Studies. (DOCX) [file pone.0222495.s003.docx]

**S3. Identified studies and reports that satisfied the inclusion criteria**

Only reports containing the most recent data were included for a given territory or country. The data were mostly reported by governmental agencies; however, four identified papers were published in scientific journals (Bartels, 2009; Flores et al., 2017; Ķipēna et al., 2013; Leonardi, 2007). Several sources (Department of Correctional Services, 2014; Department of Corrections, 2016, 2017; Ķipēna, Zavackis, & Ņikišins, 2013) did not report cohort size. Data for Denmark and Oregon, USA were obtained by using online data tools within governmental agency websites. Quality assessment was conducted using NIH Quality Assessment Tool for Observational Cohort and Cross-Sectional Studies.

| \| **Country** \| **Selection**  **period** \| **Length of**  **follow-up** \| **Cohort size** \| **Outcomes** \| **Quality assessment** \| **Source** \| \| --- \| --- \| --- \| --- \| --- \| --- \| --- \| \| **Europe** \|  \|  \|  \|  \|  \|  \| \| **Nordic countries** \|  \|  \|  \|  \|  \|  \| \| Denmark \| 2013 \| 6 months  – 2 years \| 6,501 \| Reconviction \| Good \| Statistics Denmark, 2018 \| \| Finland \| 2005 \| 2 years \| 3,767 \| Reconviction \| Good \| Graunbøl et al., 2010 \| \| Iceland \| 2005 \| 2 years \| 73 \| Reconviction \| Good \| Graunbøl et al., 2010 \| \| Norway \| 2005 \| 2 years \| 2,839 \| Reconviction \| Good \| Graunbøl et al., 2010 \| \| Sweden \| 2008 \| 1, 2, 3 years \| 22,306 \| Reconviction  (after “initial event”) \| Good \| Swedish National Council for Crime Prevention, 2017 \| \| **The United Kingdom** \|  \|  \|  \|  \|  \|  \| \| England and Wales \| 2015/2016 \| 1 year \| 139,617 \| Proven reoffending \| Good \| Ministry of Justice, 2018 \| \| Northern Ireland \| 2014/2015 \| 1-12 months \| 17,560 \| Proven reoffending \| Good \| Duncan & Damkat, 2017 \| \| Northern Ireland \| 2005 \| 2 years \| 19,047 \| Reconviction \| Good \| Department of Justice, 2011 \| \| Scotland \| 2014/2015 \| 1 year \| 21,733 \| Reconviction \| Good \| Scottish Government, 2017 \| \| **Other** \|  \|  \|  \|  \|  \|  \| \| France \| 2004 \| 5 years \| 241,996 \| Reconviction \| Good \| Ministère de la Justice, 2013 \| \| Germany \| 2010 \| 3 years \| 96,521 \| Reconviction \| Good \| Albrecht & Jehle, 2014 \| \| Italy \| 1998 \| 7 years \| 8,817 \| Reconviction \| Poor \| Leonardi, 2007 \| \| Ireland, Republic of \| 2010 \| 6 months  – 3 years \| 3,698 \| Reconviction \| Good \| Central Statistics Office, 2016 \| \| Latvia \| 2009 \| 29 months \| n/a \| Reconviction  (or initiation of proceedings) \| Fair \| Ķipēna, Zavackis, & Ņikišins, 2013 \| \| Netherlands \| 2003 \| 2 years \| 38,530 \| Reconviction  (or initiation of proceedings) \| Good \| Wartna & Tollenaar, 2006 \| \|  \|  \|  \|  \|  \|  \|  \| \| **North America** \|  \|  \|  \|  \|  \|  \| \| **Canada** \|  \|  \|  \|  \|  \|  \| \| Ontario \| 2013/2014 \| 2 years \| 35,561 \| Reconviction  (after completing a sentence) \| Good \| Ontario Ministry of Community Safety and Correctional Services, 2017 \| \| Quebec \| 2007/2008 \| 1 month  – 2 years \| 4,851 \| Reconviction  (after completing a sentence)  Reincarceration  (after completing a sentence) \| Good \| Ministère de la Sécurité publique, 2015 \| \| **USA** \|  \|  \|  \|  \|  \|  \| \| USA (federal) \| 2004/2005 \| 1-9 years \| 13,504 \| Rearrest \| Good \| Flores, Holsinger, Lowenkamp, & Cohen, 2017 \| \| Florida \| 1992/2002 \| 1, 2, 3 years \| 65,394 \| Reconviction  (for a felony) \| Good \| Bales & Piquero, 2012 \| \| Illinois \| 2006 \| 5 years \| 2,770 \| Rearrest \| Good \| Illinois Criminal Justice Information Authority, 2011 \| \| Michigan \| 2003/2006 \| 1, 3, 5 years \| 43,606 \| Reconviction  (for a felony)  Reimprisonment \| Good \| Harding et al., 2013 \| \| North Carolina \| 2013 \| 1, 2 years \| 35,103 \| Reconviction  Reincarceration  Rearrest \| Good \| Flinchum, Hevener, Hall, & Wesoloski, 2016 \| \| New York State \| 2002 \| 5 years \| 31,267 \| Reconviction \| Fair \| The Council of State Governments, 2013 \| \| Oregon \| 2014 \| 1, 2, 3 years \| 4,403 \| Reconviction  Reincarceration  Rearrest \| Good \| State of Oregon Criminal Justice Commission, 2018 \| \|  \|  \|  \|  \|  \|  \|  \| \| **South America** \|  \|  \|  \|  \|  \|  \| \| Chile \| 2007 \| 3 years \| 23,736 \| Reconviction  Rearrest \| Good \| Peillard, Correa, Chahuán, & Lacoa, 2012 \| \|  \|  \|  \|  \|  \|  \|  \| \| **Oceania** \|  \|  \|  \|  \|  \|  \| \| **Australia** \|  \|  \|  \|  \|  \|  \| \| Australia (federal) \| 2012/2013 \| 2 years \| n/a \| Reconviction \| Good \| Department of Correctional Services, 2014 \| \| New South Wales \| 2015 \| 1 year \| 16,907 \| Reconviction \| Good \| Bureau of Crime Statistics and Research, 2017 \| \| Tasmania \| 2002/2004 \| 2 years \| 347 \| Reconviction \| Good \| Bartels, 2009 \| \| West Australia \| 2012/2014 \| 2 years \| n/a \| Reconviction \| Good \| Department of Correctional Services, 2014 \| \| **New Zealand** \|  \|  \|  \|  \|  \|  \| \| New Zealand \| 2014/2015 \| 1, 2 years \| n/a \| Reconviction  Reincarceration \| Good \| Department of Corrections, 2017  Department of Corrections, 2016 \| |
| --- | --- | --- | --- | --- | --- | --- | --- | --- | --- | --- | --- | --- | --- | --- | --- | --- | --- | --- | --- | --- | --- | --- | --- | --- | --- | --- | --- | --- | --- | --- | --- | --- | --- | --- | --- | --- | --- | --- | --- | --- | --- | --- | --- | --- | --- | --- | --- | --- | --- | --- | --- | --- | --- | --- | --- | --- | --- | --- | --- | --- | --- | --- | --- | --- | --- | --- | --- | --- | --- | --- | --- | --- | --- | --- | --- | --- | --- | --- | --- | --- | --- | --- | --- | --- | --- | --- | --- | --- | --- | --- | --- | --- | --- | --- | --- | --- | --- | --- | --- | --- | --- | --- | --- | --- | --- | --- | --- | --- | --- | --- | --- | --- | --- | --- | --- | --- | --- | --- | --- | --- | --- | --- | --- | --- | --- | --- | --- | --- | --- | --- | --- | --- | --- | --- | --- | --- | --- | --- | --- | --- | --- | --- | --- | --- | --- | --- | --- | --- | --- | --- | --- | --- | --- | --- | --- | --- | --- | --- | --- | --- | --- | --- | --- | --- | --- | --- | --- | --- | --- | --- | --- | --- | --- | --- | --- | --- | --- | --- | --- | --- | --- | --- | --- | --- | --- | --- | --- | --- | --- | --- | --- | --- | --- | --- | --- | --- | --- | --- | --- | --- | --- | --- | --- | --- | --- | --- | --- | --- | --- | --- | --- | --- | --- | --- | --- | --- | --- | --- | --- | --- | --- | --- | --- | --- | --- | --- | --- | --- | --- | --- | --- | --- | --- | --- | --- | --- | --- | --- | --- | --- | --- | --- | --- | --- | --- | --- | --- | --- | --- | --- | --- | --- | --- | --- | --- | --- | --- | --- | --- | --- | --- | --- | --- | --- | --- | --- | --- | --- | --- | --- | --- | --- | --- | --- | --- | --- | --- | --- | --- | --- | --- | --- | --- | --- | --- | --- | --- | --- | --- | --- | --- | --- | --- | --- | --- | --- | --- | --- | --- | --- | --- | --- | --- | --- | --- | --- | --- | --- | --- | --- | --- | --- | --- | --- | --- |
