# Supplementary material for: Recidivism rates in individuals receiving community sentences: A systematic review
Source: PLoS One. 2019 Sep 20;14(9):e0222495. doi: 10.1371/journal.pone.0222495 (PMC6754149; doi:10.1371/journal.pone.0222495)
Supplement: S4 Table — (DOCX) [file pone.0222495.s004.docx]

**S4. Description of data extracted from the studies.**

| **Country** | **Study** | **Description of outcomes** | **Description of follow-up** | **Sentences** | **Notes and exclusions** |
| --- | --- | --- | --- | --- | --- |
| Australia –  New South Wales | Bureau of Crime Statistics and Research, 2017 | **Reconviction**  The crime and conviction should both happen during a follow-up to be counted as recidivism. Technical violations are not automatically counted as reoffence. | 1 year. Starts with an imposition of a sentence. | Any sentence other than imprisonment. | A single offender is counted only once. |
| Australia –  Tasmania | Bartels, 2009 | **Reconviction**  The crime and conviction should both happen during a follow-up to be counted as recidivism. Technical violations are not automatically counted as reoffence. | 2 years. Starts with an imposition of a sentence. | Wholly suspended sentence  Noncustodial order | Excludes pseudo-reconvictions. A single offender is counted only once. |
| Australia –  West Australia | Department of Correctional Services, 2014 | **Reconviction (return)**  Return of an individual to Corrective Services during a follow-up period. | 2 years. Starts with an end of a sentence. | Any sentence that results in an offender entering Community Corrections. | One offender may be counted several times, if he is sentenced, released and reconvicted again during a follow-up period.  Excludes returns to community correction Work and Development orders and Bail orders. Excludes fines. |
| Australia (federal) | Department of Correctional Services, 2014 | **Reconviction (return)**  Return of an individual to Corrective Services during a follow-up period. | 2 years. Starts with an end of a sentence. | Any sentence that results in an offender entering Community Corrections. | One offender may be counted several times, if he is sentenced, released and reconvicted again during a follow-up period. Excludes fines. |
| Canada –  Ontario | Ontario Ministry of Community Safety and Correctional Services, 2017 | **Reconviction**  Return to a provincial correctional supervision after committing an offence during the time of follow-up | 2 years. Start with the end of a sentence. | Community supervision | Excludes individuals sentenced to federal prisons |
| Canada –  Quebec | Ministère de la Sécurité publique, 2015 | **Reconviction**  The crime and conviction should both happen during a follow-up to be counted as recidivism**.** Technical violations are not automatically counted as reoffence | 2 years. Start with the end of a sentence. | Probation | Two types of reconviction rates are provided in the report: for a period during supervision (i.e., revocation) and during a follow-up after the end of a sentence. Taking into account recidivism during serving a sentence increases the 2-year reconviction rate to 52% |
| Chile | Peillard, Correa, Chahuán, & Lacoa, 2012 | **Rearrest**  Any rearrest during a follow-up period. In case of a successful restitution, rearrest in not counted. | 3 years. Starts with an imposition of a sentence. | Conditional sentence  Probation  Night detention |  |
| Denmark | Statistics Denmark, 2018 | **Reconviction**  3 years after follow-up ends, an individual can be sentenced for an offence committed during the follow-up period. | 2 years, broken down into 6 monthly periods. Starts with an imposition of a sentence | Suspended sentence  Community service  Withdrawal of charges with conditions | Individuals of age 19 and younger are excluded.  Online tool is provided. |
| Finland | Graunbøl et al., 2010 | **Reconviction**  The offence and conviction should both happen during a follow-up to be counted as recidivism | 2 years. Starts with an imposition of a sentence | Probation with supervision  Probation with treatment  Community service |  |
| France | Ministère de la Justice, 2013 | **Reconviction**  The offence and conviction should both happen during a follow-up to be counted as recidivism**.** | 5 years. Starts with an end of a sentence. Broken down by 1-year periods. | Conditional sentence | Two types of reconviction rates are provided in the report: for a period during supervision (i.e., revocation) and during a follow-up after the end of a sentence. Only 2.2% of a cohort were reconvicted while serving a sentence. |
| Germany | Albrecht & Jehle, 2014 | **Reconviction**  The offence and conviction should both happen during a follow-up to be counted as recidivism**.** | 3 years. Starts with an imposition of a sentence | Suspended sentence | The rates are estimated from graphs. |
| Iceland | Graunbøl et al., 2010 | **Reconviction**  The offence and conviction should both happen during a follow-up to be counted as recidivism | 2 years. Starts with an imposition of a sentence | Probation with supervision  Probation with treatment |  |
| Ireland, Republic of | Central Statistics Office, 2016 | **Reconviction**  To be counted as a recidivism event, an offence should occur within a follow-up period and a conviction should happen within two years after the offence. | 6 months – 3 years. Broken down into multiple intervals. | Probation orders  Community service orders | Sex offenders and individuals who committed certain road offences are not included in a sample. |
| Italy | Leonardi, 2007 | **Reconviction**  Operationalisation is unclear. | 7 years | Community sanctions. Exact sentences are unclear |  |
| Latvia | Ķipēna, Zavackis, & Ņikišins, 2013 | **Reconviction**  **(or initiation of proceedings)**  A new criminal charge that did not results in acquittal or other technical dismissal during a follow-up period. | 29 months. Starts with an imposition of a sentence. | Community service  Probation | The report provides two definitions of recidivism (reconviction and initiation of legal proceedings). |
| Netherlands | Wartna & Tollenaar, 2006 | **Reconviction (or initiation of proceedings)**  A new criminal charge that did not results in acquittal or other technical dismissal during a follow-up period | 2 years. Starts with an imposition of a sentence. | Community service  Training order  Conditional sentence  Discretionary dismissal | Rates for fines are reported separately (Fig. 3). |
| New Zealand | Department of Corrections, 2017  Department of Corrections, 2016 | **Reconviction**  The crime and conviction should both happen during a follow-up to be counted as recidivism | 1, 2 years. Starts with an end of an end of a sentence | Community sentence | Cohort sizes are not provided. Some offenders may be double-counted, if they participate in multiple rehabilitation programmes. |
| Norway | Graunbøl et al., 2010 | **Reconviction**  The offence and conviction should both happen during a follow-up to be counted as recidivism | 2 years. Starts with an imposition of a sentence | Probation with supervision  Probation with treatment  Community service |  |
| Sweden | Swedish National Council for Crime Prevention, 2017 | **Reconviction**  The offence and conviction should both happen during a follow-up period to be counted as recidivism | 2 years. Starts with an imposition of a sentence | Intensive supervision with electronic monitoring  Probation (incl. with community service)  Suspended sentence (incl. with community service) | One offender can be counted multiple times. For intensive supervision, follow-up starts at the end of a sentence. |
| UK – England & Wales | Ministry of Justice, 2018 | **Proven reoffending**  6 months after observational period ends, an individual can be sentenced for an offence committed during this period. | 1-year observational period. Starts with an imposition of a sentence | Pre CJA orders  Community orders  Suspended sentence order | Cautions and discharges are not included. Rates for fines are reported separately (Fig. 3). |
| UK – N. Ireland | Duncan & Damkat, 2017 | **Proven reoffending**  6 months after observational period ends, an individual can be sentenced for an offence committed during this period. Technical violations are not counted as reoffence | 1-year observational period. Starts with an imposition of a sentence | Community supervision  Community other | Diversionary disposal are not included. Rates for fines are reported separately (Fig. 3). |
| UK – N. Ireland | Department of Justice, 2011 | **Reoffending**  The offence and conviction should both happen during a follow-up period to be counted as recidivism | 2 years. Starts with an imposition of a sentence | Community service order  Probation order  Combination order  Bound over  Conditional discharge  Suspended prison  Other | Fines as an index offence are excluded in extracted overall general recidivism rate (Appendix 4). Rates reported by gender include fines as an index offence (Table 1). |
| UK – Scotland | Scottish Government, 2017 | **Reconviction**  The conviction should happen during a follow-up period to be counted as recidivism | 1-year. Starts with an imposition of a sentence | Restriction of liberty order  Community payback order  Drug treatment and testing orders | Rates for fines are reported separately (Fig. 3). An offence against public justice are not counted as an index offence. |
| USA – Illinois | Illinois Criminal Justice Information Authority, 2011 | **Rearrest**  An arrest during a follow-up period. | 5 years. Starts with an imposition of a sentence. | Probation | Data are reported separately for a period during probation and after probation. Combined rate was extracted. Minor traffic offences are excluded. |
| USA – Michigan | Harding et al., 2013 | **Reincarceration**  New sentence that leads to imprisonment during a follow-up period  **Reconviction**  New sentence for a felony during a follow-up period | 1, 3, 5 years. Starts with an imposition of a sentence. | Probation | The cohort includes individuals sentenced for a felony. Separate data on reincarceration resulting from technical violation and from a new sentence are provided. |
| USA – New York State | The Council of State Governments, 2013 | **Reconviction**  Operationalisation is unclear | 5 years. Starts with an imposition of a sentence. | Probation | For all felony probation sentences in NYS, 5 years is also a supervision period. |
| USA – North Carolina | North Carolina Sentencing and Policy Advisory Commission, 2016 | **Rearrest**  **Reconviction Reincarceration**  An occurrence of a respective event during a follow-up period. Arrests for technical violations are not counted as recidivism. Only rearrests when an individual was fingerprinted | 1, 2 years. Starts with an imposition of a sentence. | Probation | Excludes offenders with serious DWI, serious misdemeanor traffic offence. |
| USA – Oregon | State of Oregon Criminal Justice Commission, 2018 | **Reconviction**  **Rearrest**  **Reincarceration**  An occurrence of a respective event during a follow-up period. Only rearrests when an individual was fingerprinted. | 1, 2, 3 years. Starts with an imposition of a sentence. | Probation | Online tool is provided.  One offender can be counted multiple times, since each new admission is considered a separate case.  Sample does not include those sentenced to felony bench or court probation. |
| USA (federal) | Flores, Holsinger, Lowenkamp, & Cohen, 2017 | **Rearrest**  An arrest during a follow-up period. | 1-9 years. Broken down into multiple intervals. | Probation | Rates are based on random samples that consist of offenders that had undertaken PCRA assessment |

**List of identified sources for Appendix 3**

Albrecht, H.-J. & Jehle, J.-M. eds., 2014. National Reconviction Statistics and Studies in Europe = Nationale Rückfallstatistiken und-untersuchungen in Europa. Göttinger Studien zu den Kriminalwissenschaften. Available from: <http://dx.doi.org/10.17875/gup2014-791>.

Bartels, L. (2009). The weight of the sword of Damocles: A reconviction analysis of suspended sentences in Tasmania. The Australian and New Zealand Journal of Criminology 42(1): 72--100.

Bureau of Crime Statistics and Research. (2017). Re-offending​ statistics for NSW. Retrieved 15 Jun 2018. <http://www.bocsar.nsw.gov.au/Pages/bocsar_pages/Re-offending.aspx>

Central Statistics Office. (2016). Probation recidivism 2010 cohort. Retrieved 1 May 2018 from: <http://www.cso.ie/en/releasesandpublications/er/pror/probationrecidivism2010cohort/>

Department of Correctional Services. (2014). Recidivism trends in Western Australia with comparison to national trends. Retrieved 15 Jun 2018: <https://www.correctiveservices.wa.gov.au/_files/about-us/statistics-publications/statistics/DCS-recidivism-trends-WA-October2014.pdf>

Department of Corrections. (2016). Annual report: 1 July 2015 - 30 June 2016. Retrieved 15 June 2018 from: <http://www.corrections.govt.nz/__data/assets/pdf_file/0010/857737/Annual_report_201516.pdf>

Department of Corrections. (2017). Annual report: 1 July 2016 - 30 June 2017. Retrieved 15 June 2018 from: <http://www.corrections.govt.nz/__data/assets/pdf_file/0006/898629/Annual_Report_2016-17.pdf>

Department of Justice. (2011). Adult reconviction in Northern Ireland 2005, Statistics and Research Branch, Department of Justice, Belfast. Retrieved 1 May 2018 from: <https://www.justice-ni.gov.uk/sites/default/files/publications/doj/adult-reconviction-in-northern-ireland-2005.pdf>

Duncan, L., and Damkat, I. (2017). Adult and youth reoffending in Northern Ireland (2014/15 Cohort), Analytical Service Group, Department of Justice, Belfast. Retrieved from: <https://www.justice-ni.gov.uk/sites/default/files/publications/justice/r-bulletin-29-2017-adult-and-youth-reoffending-northern-ireland-201415-cohort.pdf>

Flinchum, T., Hevener, H., Hall, M., & Wesoloski, J. (2016). Correctional Program Evaluation: Offenders Placed on Probation or Released from Prison in FY 2013, North Carolina Sentencing and Policy Advisory Commission, Raleigh, NC. Retrieved 1 May 2018 from: <https://www.nccourts.gov/assets/documents/publications/recidivism_2016.pdf>

Flores, A. W., Holsinger, A.M., Lowenkamp, C.T., and Cohen, T.H. (2017). Time-free effects in predicting recidivism using both fixed and variable follow-up periods: Do different methods produce different results. Crim Just Beh 44(1): 121--137.

Graunbøl H.M., Kielstrup B., Muiluvuori M.-L., Tyni S., Baldursson E.S., Gudmundsdottir H., et al. (2010). Retur: en nordisk undersøgelse af recidiv blant klienter i kriminalforsorgen Oslo: Kriminalomsorgens utdanningssenter. Retrieved 15 April 2018 from: <http://www.kriminalforsorgen.dk/Files/Filer/Statistik/Retur_-_nordisk_recidiv_maj_2010.pdf>

Harding, D.J., Morenoff, J.D., Nguyen, A.P., and Bushway, S.D. (2017). Short- and long-term effects of imprisonment on future felony convictions and prison admissions. Proceedings of the National Academy of Sciences, 114(42): 11103--11108. doi:10.1073/pnas.1701544114

Illinois Criminal Justice Information Authority. (2011). Examining Illinois probationer characteristics and outcomes. Retrieved 1 May 2018 from: <http://www.icjia.state.il.us/assets/pdf/ResearchReports/Examining_IL_probationer_characteristics_and_outcomes_092011.pdf>

Ķipēna, K., Zavackis A., and Ņikišins J. (2013). Sodu izcietušo personu noziedzīgo nodarījumu recidīvs. Jurista Vārds, 35(786): 12--17.

Leonardi, F. (2007). Le misure alternative alla detenzione tra reinserimento sociale e abbattimento della recidiva. Rassegna penitenziaria e criminological, 2: 7--26.

Ministère de la Justice (2013). Mesurer la récidive: Contribution à la conférence de consensus de prévention de la récidive. Retrieved 1 June 2018 from: <http://www.justice.gouv.fr/art_pix/stat_recidive_2013.pdf>

Ministère de la Sécurité publique. (2015). Projet: Enquête sur la récidive/reprise de la clientèle confiée aux Services correctionnels du Québec. Retrieved 1 May 2018 from: <https://www.securitepublique.gouv.qc.ca/services-correctionnels/publications-et-statistiques/enquete-sur-la-recidivereprise.html>

Ministry of Justice. (2018). Proven reoffending statistics quarterly: January 2016 to March 2016. Retrieved 1 May 2018 from: <https://www.gov.uk/government/statistics/proven-reoffending-statistics-january-2016-to-march-2016>

Ontario Ministry of Community Safety and Correctional Services. (2017). Rates of recidivism (re-conviction) in Ontario. Retrieved 15 May 2018 from: <https://www.mcscs.jus.gov.on.ca/english/Corrections/RatesRecidivism.html>

Peillard, A.M.M., Correa, N.M., Chahuán, G.W., and Lacoa, J.F. (2012). La Reincidencia en el Sistema Penitenciario Chileno, Santiago. Retrieved 1 June 2018 from: <http://www.pensamientopenal.com.ar/system/files/2017/09/doctrina45811.pdf>

Scottish Government. (2017). Reconviction Rates in Scotland: 2014-15 Offender Cohort. Retrieved 1 May 2018 from: <https://www.gov.scot/Publications/2017/05/8362/0>

State of Oregon Criminal Justice Commission. (2018). Recidivism. Retrieved 1 May 2018 from: <http://www.oregon.gov/cjc/data/Pages/recidivism.aspx>

Statistics Denmark. (2018). Recidivism. Retrieved 1 May 2018 from: <https://www.dst.dk/en/Statistik/emner/levevilkaar/kriminalitet/tilbagefald-til-kriminalitet>t

Swedish National Council for Crime Prevention. (2017). Recidivism. Retrieved 1 May 2018 from: <https://www.bra.se/bra-in-english/home/crime-and-statistics/crime-statistics/recidivism.html>

The Council of State Governments. (2013). Improving probation and alternatives to incarceration in New York State: Increasing public safety and reducing spending on prisons and jails. Retrieved 1 May 2018 from: <https://csgjusticecenter.org/wp-content/uploads/2013/03/122112_Probation-ATI-Recs_BRIEF_for-NYSAC.pdf>

Wartna, B.S.J., and Tollenaar, N. (2006). Recidive 1997-2003: Ontwikkelingen in het niveau van de strafrechtelijke recidive van jeugdige en volwassen daders, Wetenschappelijk Onderzoeken Documentatiecentrum, Den Haag.
