# Supplementary material for: Recidivism rates in individuals receiving community sentences: A systematic review
Source: PLoS One. 2019 Sep 20;14(9):e0222495. doi: 10.1371/journal.pone.0222495 (PMC6754149; doi:10.1371/journal.pone.0222495)
Supplement: S5 Table — (DOCX) [file pone.0222495.s005.docx]

**S5. Recent studies in community sentenced populations that utilised advanced research designs**

Search on SAGE Journals (January 1, 2014 to July 20, 2019) with no language restrictions. References of screened-in papers were scanned. We included randomised trials and matched cohort studies in adult individuals receiving community sentences. The studies based on self-reported outcomes were excluded.

Search terms: [[All 'community sentence'] OR [All probation]] AND [All recidivism] AND [[All matching] OR [All randomi*]]

| Publication | Research question | Sample | Research design | Primary outcome | Main results |
| --- | --- | --- | --- | --- | --- |
| Bales & Piqeuro, 2012 | The impact of custodial sentences on reoffending | 144,416 offenders sentenced to prison or intensive supervision in Florida, USA | Propensity score matching, exact score matching | Reconviction for a felony during 3 years after release (prisoners) or receiving a sentence (community orders) | *3-year reconviction rates (precision/exact matching)*  *Note: rates are depended on matching models*  Released from custody: 18.2% - 49.1%  Community orders: 15.4% - 38.4%  *3-year reconviction rates (propensity score matching)*  *Note: rates are depended on matching models*  Released from custody: 42.9% - 53.8%  Community orders: 31.3% - 34.8% |
| DeVall et al., 2017 | The impact of the Swift and Sure Sanctions Probation Programme (SSSPP) on reoffending | 758 offenders sentenced to either regular probation or SSSPP in Michigan, USA | Propensity score matching | Charge for a new offence after the allocation to the programme (maximum 23 months of flexible follow-up) | *New charge rates*  SSSPP group: 37.7%  Comparison group: 46.7% |
| Evans et al., 2014 | The impact of the Proposition 36 programme on reoffending | 29,321 drug offenders referred to drug courts or Proposition 36 programme in California, USA. Includes individuals on parole | Propensity score matching | Posttreatment re-arrest during 12 months after the completion of the treatment | *1-year re-arrests rates from (unmatched cohorts)*  Prop 36: 48.0%  Drug courts: 44.0%  *1-year rearrests rates from (matched cohorts)*  Prop 36: 49.7%  Drug courts: 43.1% |
| Hyatt & Barnes, 2014 | The impact of Intensive Supervision Probation (ISP) on probationer recidivism | 832 high-risk offenders under the community supervision in Philadelphia, USA | Random forest forecasting | Charge for a new offence during the 12 months following the allocation to the programme | *New charge rates*  ISP group: 40,5%  Comparison group: 41.6%  *Detected violations*  ISP group: 43,0%  Comparison group: 27.0% |
| Jollife and Hedderman, 2015 | The impact of custodial sentences on reoffending | 5,500 male offenders from the United Kingdom | Nearest neighbour matching and stratification based on propensity scores | Reconviction after 12 months after release (prisoners) or receiving a sentence (community orders) | *1-year reconviction rates in matched samples*  Released from custody: 51.1%  Community orders: 44.5% |
| Lowenkamp et al., 2014 | The impact of STARR training programme for probation officers | 999 offenders on post-conviction supervision from the USA (unspecified state) | Random assignment of probation officer to training programmes | Re-arrest during 24 months following the initial conviction | *2-year re-arrest rates for all offenders*  STARR trained: 43.0%  Control: 48.0%  *2-year re-arrest rates for moderate risk offenders*  STARR trained: 41.0%  Control: 28.0%  *2-year re-arrest rates for high risk offenders*  STARR trained: 55.0%  Control: 55.0% |
| Pearson et al., 2016 | The impact of the ‘Citizenship’ probation supervision program on recidivism | 1,091 offenders with medium to high risk of reconviction from England and Wales | Stepped wedge cluster randomisation | Time to reconviction during the period of 6-18 months from the allocation. | *1-year reoffending rates estimated from survival* curves ≈46.0% in both groups.  *Note: no crude rates reported. Some reduction in adjusted reoffending rates in the intervention group (no statistically significant results).* |
| Quinn and Quinn, 2015 | The impact of cognitive-behavioural therapy programme on reoffending | 286 defendants on probation supervision with histories of repeated driving while intoxicated offences from New York State, USA | Samples matched by distribution of demographic characteristics | Charge for a serious traffic offence during 3 years from the allocation | *New charge rates*  CBT group: 11.0%  Comparison group: 24.0% |
| Sorsby et al., 2017 | The impact of Skills for Effective Engagement and Development (SEED) training programme for case officers on community orders compliance | 931 individuals receiving community orders from 3 different probation trusts in England and Wales | Regression adjustment based on 1. treatment covariates;  2. propensity scores. | Compliance with community orders (completion) | *Unadjusted non-completion rates*  SEED trained: 25,1%  Control: 27,7%  No matching or randomisation were used during the allocation. |
| Trevena and Weatherburn, 2015 | The impact of short custodial sentences on reoffending | 7,920 individuals sentenced to short-term (up to 12 months) imprisonment or receiving suspended sentences (up to 2 years) in New South Wales, Australia | Propensity score matching | Time to reconviction during the period of up to 6 years from the allocation. | *3-year reconviction rates (unmatched)*  Released from custody: 45.6%  Suspended sentence: 39.7%  *3-year reconviction rates (propensity score matching)*  Released from custody: 43.4%  Suspended sentence: 42.3% |

**List of identified sources for Appendix 4**

Bales, W.D. & Piquero, A.R. (2012). Assessing the impact of imprisonment on recidivism. Journal of Experimental Criminology, 8:71-101. <https://doi.org/10.1007/s11292-011-9139-3>

Christopher T. Lowenkamp, Alexander Holsinger, Charles R. Robinson & Melissa Alexander (2014) Diminishing or durable treatment effects of STARR? A research note on 24-month re-arrest rates, Journal of Crime and Justice, 37:2, 275-283, DOI: 10.1080/0735648X.2012.753849

DeVall, K. E., Lanier, C., Hartmann, D. J., Williamson, S. H., & Askew, L. N. (2017). Intensive Supervision Programs and Recidivism: How Michigan Successfully Targets High-Risk Offenders. The Prison Journal, 97(5), 585–608. <https://doi.org/10.1177/0032885517728876>

Evans, E., Li, L., Urada, D., & Anglin, M. D. (2014). Comparative Effectiveness of California’s Proposition 36 and Drug Court Programs Before and After Propensity Score Matching. Crime & Delinquency, 60(6), 909–938. <https://doi.org/10.1177/0011128710382342>

Hyatt, J. M., & Barnes, G. C. (2017). An Experimental Evaluation of the Impact of Intensive Supervision on the Recidivism of High-Risk Probationers. Crime & Delinquency, 63(1), 3–38. <https://doi.org/10.1177/0011128714555757>

Jolliffe, D., & Hedderman, C. (2015). Investigating the Impact of Custody on Reoffending Using Propensity Score Matching. Crime & Delinquency, 61(8), 1051–1077. <https://doi.org/10.1177/0011128712466007>

Pearson, D. A. S., McDougall, C., Kanaan, M., Torgerson, D. J., & Bowles, R. A. (2016). Evaluation of the Citizenship Evidence-Based Probation Supervision Program Using a Stepped Wedge Cluster Randomized Controlled Trial. Crime & Delinquency, 62(7), 899–924. <https://doi.org/10.1177/0011128714530824>

Quinn, T. P., & Quinn, E. L. (2015). The Effect of Cognitive-Behavioral Therapy on Driving While Intoxicated Recidivism. Journal of Drug Issues, 45(4), 431–446. <https://doi.org/10.1177/0022042615603390>

Sorsby, A, Shapland, J, Robinson, G (2017) Using compliance with probation supervision as an interim outcome measure in evaluating a probation initiative. Criminology and Criminal Justice 17(1): 40–61.

Trevena, J., & Weatherburn, D. (2015). Does the first prison sentence reduce the risk of further offending? Contemporary Issues in Crime and Justice, 187. Available from: <https://www.bocsar.nsw.gov.au/Documents/CJB/Report-2015-Does-the-first-prison-sentence-reduce-the-risk-of-further-offending-cjb187.pdf>
