# Supplementary material for: Recidivism rates in individuals receiving community sentences: A systematic review
Source: PLoS One. 2019 Sep 20;14(9):e0222495. doi: 10.1371/journal.pone.0222495 (PMC6754149; doi:10.1371/journal.pone.0222495)
Supplement: S6 Table — (DOCX) [file pone.0222495.s006.docx]

| **COUNTRY:** |  |
| --- | --- |
| **Population:** | **Imprisoned - Convicted – Arrested – Given a community sentence** |
| **Outcome:** | **Reimprisonment - Reconviction – Rearrest – Community sentence revocation** |
| **Follow-up:** | **1 year - 2 years - 3 years - 4 years - 5 years** |
| **Rate:** | **%** |

**DETAILED INFORMATION**

| **POPULATION** | **NOTES** | **RECOMMENDED** | **P#** |
| --- | --- | --- | --- |
| **1. Inclusion criteria** |  |  |  |
| **a. Geographical** | Are you reporting on the country as a whole, or a specific region? | Include whole country. |  |
| **b. Index disposal** | Who is included? Imprisoned, convicted, arrested, receiving community sentences? | Imprisonment and/or community sentences. Provide clear description of each type of community sentence. |  |
| **c. Index offence** | Define offenses and provide clear description of any offence groupings (e.g., against property, violent, etc.) |  |  |
| **d. Population** | What institutions? Sub-sample or whole country? | All adult prisoners (age 18+) and/or community sentenced population. Report sample size. |  |
| **e. Time period** | When did the observation period start? | Date of release from prison. For community sentences use two observational periods: during the sentence, and starting from the date of sentence completion. |  |
| **2. Exclusion criteria** |  |  |  |
| **a. Other samples** | Who is not included? e.g. secure hospital patients, youth institutions. | Exclude individuals sent to secure hospital, prisoners and/or individuals receiving community sentences under 18. |  |
| **b. Other oﬀences** | What is not included? e.g. non-indictable offences/misdemeanours, minor offences, antisocial behaviour. | Include all prisoners and/or individuals receiving community sentences. If any exclusions, report estimated numbers. |  |
| **c. Other disposals** | What is not included? e.g. fines, electronic monitoring, mandatory treatment, other sanctions. | Include all prisoners and/or individuals receiving community sentences. If any exclusions, report estimated numbers. |  |
| **3. Sample** |  |  |  |
| **a. Basic characteristics** | Age, gender, ethnicity, education level. | Gender. 10-year age bands (18-24, 25-34, 35-44, 45-54,  55-64, 65+). |  |
| **b. Index oﬀences** | What offences had been committed in the population? | Burglary/Theft, Fraud, Sexual offences (incl. rape), Violence against person, Motoring, Drugs, Other, not recorded. |  |
| **c. Disposal** | If prisoners, what length of time did they stay?  If individuals receiving community sentences, what disposal and what is the length of sentence? | <1 year, <5 years, <10 years, ≧10 years.  Report data separately for each included sentenced type (imprisonment, community service, electronic monitoring, mandatory training, treatment and rehabilitation, movement restriction, others) |  |
| **OUTCOME** | **NOTES** | **RECOMMENDED** | **P#** |
| **1. Inclusion criteria** |  |  |  |
| **a. Geographical** | Where can the recidivism occur? | Whole country. |  |
| **b. Outcome disposal** | Definition of recidivism? Imprisoned, convicted, arrested? | Convicted of any offence and recalls. Any revocation of a community sentence. |  |
| **c. Follow-up** | How long was the sample followed-up for? | 2 year follow-up (or more follow-up periods) after sentence is completed. For community sentences, also use the length of a sentence. |  |
| **d. Censoring** | How was the follow-up data censored? e.g. by using survival analysis. |  |  |
| **d. Date** | What date was used? e.g. date of offence, date of conviction. | Use date of offence. |  |
| **2. Exclusion criteria** |  |  |  |
| **a. Other oﬀences** | What is not included? e.g. traffic offences, non-indictable offences/misdemeanours, etc. | Exclude minor traffic offences only. |  |
| **b. Other disposal** | What is not included? e.g. fines, community service, suspended sentence. | Include all convictions. Exclude cautions. |  |
|  |  |  |  |
| **DATA** | **NOTES** | **RECOMMENDED** | **P#** |
| **1. Source** |  |  |  |
| **a. Population** | Where was the population sample data collected from? | National agencies. |  |
| **b. Outcome** | Where was outcome data collected from? | National crime agency. |  |
| **2. Quality** |  |  |  |
| **a. Missing data** | Loss to follow-up, underreporting, misclassification etc. |  |  |
| **b. Linkage** | How were offences to those released/convicted? (e.g. date of birth). | Unique identifiers. |  |
| **c. Pseudoconvictions** | How were new convictions which related to offences before the index offence treated? | Exclude. |  |
| **3. Funding** | Who funded the data linkage and research in general? |  |  |
